# Resistance to *Plasmopara viticola *in a grapevine segregating population is associated with stilbenoid accumulation and with specific host transcriptional responses

**DOI:** 10.1186/1471-2229-11-114

**Published:** 2011-08-12

**Authors:** Giulia Malacarne, Urska Vrhovsek, Luca Zulini, Alessandro Cestaro, Marco Stefanini, Fulvio Mattivi, Massimo Delledonne, Riccardo Velasco, Claudio Moser

**Affiliations:** 1 Fondazione Edmund Mach, Research and Innovation Center, Via E.Mach 1, 38010 San Michele all'Adige, Italy; 2 Department of Biotechnology, University of Verona, Strada le Grazie 15, 37134 Verona, Italy

## Abstract

**Background:**

Downy mildew, caused by the oomycete *Plasmopara viticola*, is a serious disease in *Vitis vinifera*, the most commonly cultivated grapevine species. Several wild *Vitis *species have instead been found to be resistant to this pathogen and have been used as a source to introgress resistance into a *V. vinifera *background. Stilbenoids represent the major phytoalexins in grapevine, and their toxicity is closely related to the specific compound. The aim of this study was to assess the resistance response to *P. viticola *of the Merzling × Teroldego cross by profiling the stilbenoid content of the leaves of an entire population and the transcriptome of resistant and susceptible individuals following infection.

**Results:**

A three-year analysis of the population's response to artificial inoculation showed that individuals were distributed in nine classes ranging from total resistance to total susceptibility. In addition, quantitative metabolite profiling of stilbenoids in the population, carried out using HPLC-DAD-MS, identified three distinct groups differing according to the concentrations present and the complexity of their profiles. The high producers were characterized by the presence of *trans*-resveratrol, *trans*-piceid, *trans*-pterostilbene and up to thirteen different viniferins, nine of them new in grapevine.

Accumulation of these compounds is consistent with a resistant phenotype and suggests that they may contribute to the resistance response.

A preliminary transcriptional study using cDNA-AFLP selected a set of genes modulated by the oomycete in a resistant genotype. The expression of this set of genes in resistant and susceptible genotypes of the progeny population was then assessed by comparative microarray analysis.

A group of 57 genes was found to be exclusively modulated in the resistant genotype suggesting that they are involved in the grapevine-*P. viticola *incompatible interaction. Functional annotation of these transcripts revealed that they belong to the categories defense response, photosynthesis, primary and secondary metabolism, signal transduction and transport.

**Conclusions:**

This study reports the results of a combined metabolic and transcriptional profiling of a grapevine population segregating for resistance to *P. viticola*. Some resistant individuals were identified and further characterized at the molecular level. These results will be valuable to future grapevine breeding programs.

## Background

The cultivated European *Vitis vinifera L*. produces high quality grapes but is prone to several diseases. However, other species of the genus *Vitis*, originally from Eastern Asia and North and Central America, have been described as partially or totally resistant to several pathogens [1-4]. Among these, the oomycete *Plasmopara viticola *(Berk. and Curt.) Berl. and de Toni is a major problem for grapevine production around the world. In susceptible cultivars, this biotrophic pathogen rapidly invades infected leaves causing yellowish oily spots on the upper leaf surface and massive sporulations on the underside [5]. Invasion also occurs in resistant genotypes, but proliferation is swiftly blocked by a combination of constitutive and post-infection resistance mechanisms [6,7].

Indeed, resistant *Vitis *species may benefit from a higher level of constitutive resistance to *P. viticola *[8-10] and display post-infection resistant mechanisms which trigger the accumulation of reactive oxygen species, antimicrobial phenolic compounds, as well as pathogenesis-related proteins and peroxidases [3,11-13]. These events lead to morphological changes in the cell, including cell-wall thickening, necrosis and in some cases localized hypersensitive response (HR) [12,14,15].

Stilbenoids represent the major antimicrobial phenolic compounds in grapevine [16-19], and they may be constitutively expressed in the lignified organs [20-22] and in the grapes [23], or they may be elicited by fungal infection [17], abiotic stresses or elicitors [24-27].

The complex genetic basis of the resistance mechanisms of grapevine against *P. viticola *have been extensively investigated both by quantitative trait loci (QTL) analysis of segregating populations and by genome-wide expression studies comparing resistant and susceptible species. QTL studies have identified a few major resistance loci [28-32] which are particularly rich in resistance gene analogs (RGAs). Transcriptomic analyses of compatible and incompatible interactions in grapevine [6,33,34] emphasized the complexity of plant response and highlighted modulation of a large fraction of the entire transcriptome in both cases, although this occurs earlier and with greater intensity in the incompatible interaction.

In the present work we investigated the variability in resistance to *P. viticola *of the Merzling (M) × Teroldego (T) cross by assaying the stilbenoid profile of the entire population and the transcriptomic differences between resistant and susceptible individuals following *P. viticola *infection. This study is part of a wider survey of the mechanisms of resistance to *P. viticola *in the M × T cross, which included isolation and structural characterization of all viniferins [35] and validation of a novel method of analysis by HPLC-DAD-MS for quantification of them in infected grapevine leaves [36].

## Results

### Segregation of the *P. viticola-*resistant phenotype and stilbenoid content in the progeny population

**Figure 1:**
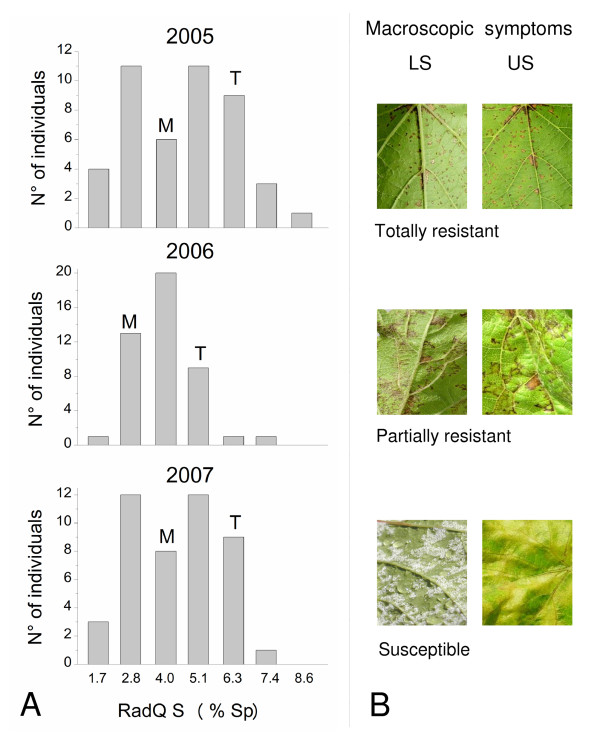
**Characterization of the resistance trait in the Merzling × Teroldego cross in three vintages**. A) Distribution of progeny from Merzling × Teroldego based on the percentage area of sporulation (% Sp) on the lower side of leaves, square root transformed (RADQ). A total of 45 individuals from all three years were considered for the distribution analysis. Values of the parents Merzling (M) and Teroldego (T) are indicated on *top *of the corresponding histogram. B) Macroscopic symptoms on lower side (LS) and upper side (US) of the leaves upon fungal infection at 10 days post *P. viticola *infection.

A continuous variation in sensitivity to *P. viticola*, taken as the percentage area of sporulation (% Sp) on the lower leaf surface, was found in the M × T population in all the infection experiments performed in the three different years (Figure 1A). The two tails of the distribution were populated by individuals displaying total resistance on one side and by completely susceptible individuals on the other side. The former were characterized by small necrotic HR spots and absence of sporulation, whereas the latter exhibited diffuse chlorosis, yellowish oily spots and high sporulation (Figure 1B).

Comparison of the distributions for the three years highlighted a general conservation in the range of variation in the observed phenotype, and differences in the frequencies of the phenotypic classes. This phenotype appears to be dependent on environmental factors. In particular, in 2005 and 2007 the square root transformed % Sp values (RADQ S) of the progeny had a bimodal distribution, while in 2006 the central classes were more populated giving the distribution a normal trend.

Assessment of sensitivity to downy mildew using the OIV452 descriptor [37], which takes into account all the plant symptoms instead of just the area of sporulation, found individuals to be distributed in nine classes ranging from total resistance to total susceptibility (Additional file 1).

The parents in all three years, one confirmed to be partially resistant (M) and the other susceptible (T), showed a certain degree of variability regardless of the severity of the symptoms. Interestingly, the range of sensitivity to *P. viticola *identified in the segregating population was greater than that delimited by the parents, suggesting transgressive segregation of the resistance trait.

An improved version [36] of a previous method [38,39] was used to measure stilbenoid accumulation in the infected leaves of the 106 individuals in a pooled sample of the second and third leaves of the shoot. Following analysis of the total stilbenoid content at 6 days post infection (dpi), individuals of the population were classified into three distinct groups (Additional file 1). The high producers (18 individuals) had the highest total stilbenoid content with an average of 78.8 μg/g fresh weight (fw) and a range of 146.3 μg/g fw to 19.8 μg/g fw). The second group, low producers, was the largest (66 individuals) with an average total stilbenoid content of 2.7 μg/g fw (range 15.4 μg/g fw to 0.2 μg/g fw). The remaining 22 individuals were considered non-stilbenoid producers, concentrations being below the quantification limit.

At 6 dpi, we were able to identify 3 monomeric stilbenes and 13 stilbenoid viniferins in the high producer group, including dimers, trimers and tetramers of resveratrol. Some of them, such as *trans*-resveratrol, *trans*-piceid, *trans*-pterostilbene, (+)-*E*-ε-viniferin, α-viniferin, *E*-miyabenol C and pallidol have already been found in grapevine and have in some cases been linked to the plant's response to fungal attack [18,39-41]. In addition, we were able to identify and quantify other viniferins (ampelopsin D, quadrangularin A, *Z*- and *E*-ω-viniferin, *Z*- and *E-*miyabenol C, isohopeaphenol, ampelopsin H and vaticanol-C-like isomer) as yet undiscovered in grapevine and which may contribute to *P. viticola *resistance. These compounds have been isolated and structurally characterized by Mattivi *et al*. [35]. The relative quantities of the different stilbenoids varied considerably, isohopeaphenol being the most abundant (between 2.6 and 68.4 μg/g fw) and *Z*- and *E*-ω-viniferin the least (below 1.25 μg/g fw). Their distribution within the high stilbenoid producers was also highly variable, suggesting that stereospecific oxidation reactions led to different patterns of viniferins in the infected leaves of different genotypes (Additional file 2).

**Figure 2:**
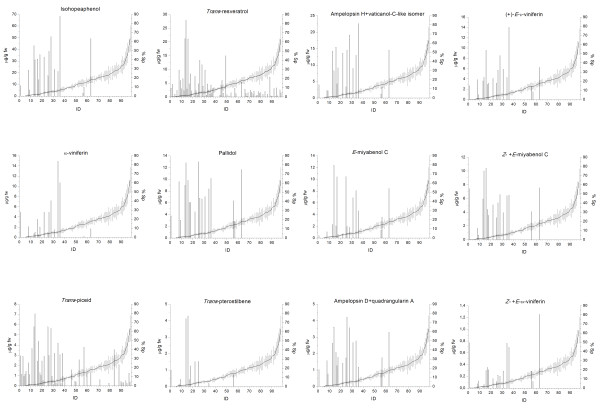
**Stilbenoid profiling of the Merzling × Teroldego cross**. Double-y plots of the concentrations (μg/g fw) of the 16 stilbenoids in infected leaves of the 106 individuals of the Merling (M) × Teroldego (T) cross (first y axis) and the percentage area of sporulation (% Sp) (second y axis). Individuals, whose codes are described in Additional file 1, were ordered on the basis of the percentage area of sporulation (% Sp) on the lower side of leaves. Biochemical and phenotypic data were available for a total of 96 individuals. ID: numeric code assigned to each genotype listed in Additional file 1.

It is also evident from Figure 2 that there is a negative correlation between the content of the different stilbenoids and the percentage of sporulation observed following infection. With very few exceptions, the high producers were also the individuals with the least severe sporulation symptoms. This does not hold true in the case of the monomeric stilbenes *trans*-resveratrol and *trans*-piceid, which were also found in the individuals with high sporulation and were the only stilbenes detected in the low producers (Figure 2 and Additional file 1).

### Gene expression analysis of resistant and susceptible genotypes

Phenotypic and metabolic profiling of the progeny population showed a positive correlation between the offsprings' resistance to *P. viticola *and the stilbenoid content of their leaves. To further investigate the plants' resistance response to *P. viticola*, we took advantage of one transgressive genotype (F1 21/66) showing almost total resistance and a high content of stilbenoids. The F1 21/66 genotype and its resistant parent Merzling were subjected to cDNA-AFLP analysis at different times following infection. The expression profile of the *P. viticola*-responsive genes was then validated by a targeted microarray analysis, which also allowed us to compare the expression response of the F1 21/66 genotype versus two susceptible ones (Teroldego and F1 22/73).

### cDNA-AFLP analysis

A cDNA-AFLP analysis was performed to study the transcriptional changes occurring during resistance response to *P. viticola *in the almost totally resistant offspring 21/66 and in the partially resistant parent Merzling.

**Figure 3:**
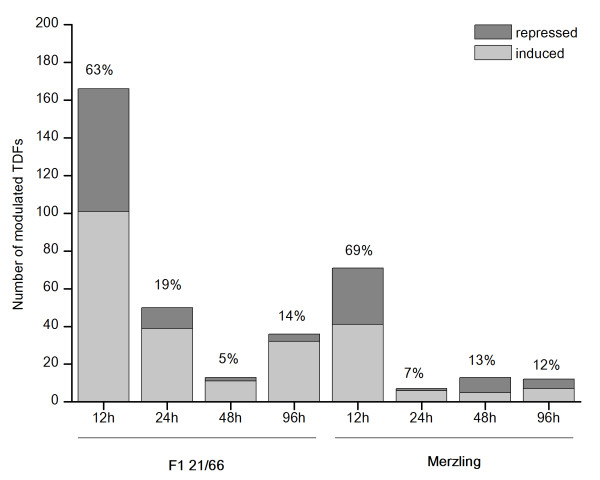
**Transcripts modulated by infection with P. viticola revealed by cDNA-AFLP analysis**. Piled histograms representing the number of transcript derived fragments (TDFs), induced (light gray) and repressed (dark gray), in F1 21/66 and in Merzling at 12, 24, 48, 96 hpi with *P. viticola*. The total percentage of modulated fragments for each time point is shown above each bar. The complete list of TDFs is available in Additional file 3.

The expression of approximately 7,000 transcript-derived fragments (TDFs) was monitored using 128 different BstYI+1/MseI+2 primer combinations (PCs) for selective amplification. We were able to visualize 55 to 75 fragments, 50-1000 bp in size, for each PC. Four hundred TDFs showed a modulated expression profile upon infection by comparing the intensity of the bands in treated samples (12, 24, 48, 96 hours post infection-hpi) with those in controls (0 hours post mock-inoculation-hpmi). Interestingly, 272 (68%) of the 400 TDFs were modulated only in the F1 21/66 genotype and not in the parent Merzling. Moreover, the kinetics of the modulation of the 400 transcripts differed. Two major gene expression patterns were predominant in both genotypes: a large group of early modulated genes which appear to be switched on within 12 hpi (63% in F1 21/66 and 69% in Merzling) and a group of late activated genes which were modulated from 48 hpi (19% in F1 21/66 and 15% in Merzling). The fraction of induced TDFs was generally much larger than the repressed ones in both groups; this tendency was more evident for the late genes of the resistant offspring (Figure 3).

The differentially-expressed fragments were excised from the gel and re-amplified by PCR using the appropriate selective PCs (data not shown). The PCR products yielded 278 good quality unique sequences (70%). Of the 278 TDFs, 265 were modulated in F1 21/66 and 103 in Merzling. The remaining sequences were not unique and could not be attributed unambiguously, probably because of two or more co-migrating fragments.

**Figure 4:**
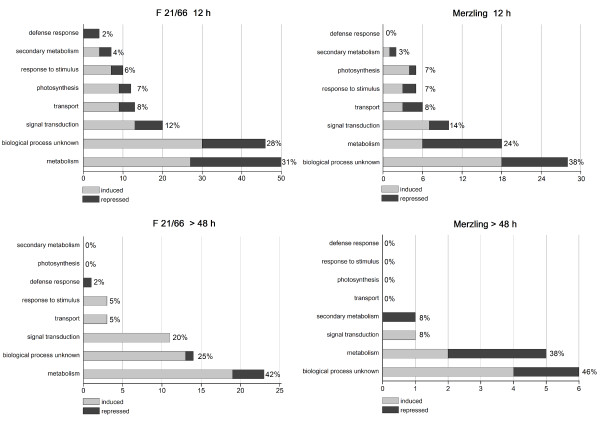
**Functional categories of transcripts modulated in F1 21/66 and in Merzling upon infection with *P. viticola***. Transcripts modulated in F1 21/66 and in Merzling within 12 hpi and after 48 hpi were assigned to 8 functional categories on the basis of automatic annotation manually revised in light of evidence from the literature. Induced genes are represented in light gray, repressed genes in dark gray. The total percentage of TDFs within each class is shown next to each bar. Details of the annotation are given in Additional file 3. In both cases, each TDF was counted only once when modulated at more than one time point.

Of the 278 sequences, 261 matched with a database and were functionally annotated (Additional file 3). The remaining 17 sequences did not match any significant database nor the known *Phytophthora *spp. sequences derived from the *Phytophthora *genome sequence [42]. Automatic annotation of the 278 transcripts was performed using the Gene Ontology (GO) classification [43] and this was then further curated manually. TDFs were assigned to 8 GO functional categories, with distinctions made between early- and late-modulated transcripts and between the two genotypes, as depicted in Figure 4.

Primary metabolism was the largest category in both genotypes, followed by signal transduction, transport, photosynthesis and response to stimulus. Interestingly, genes of the defense response and secondary metabolism classes were more highly modulated in the resistant genotype, mostly occurring in the first 24 hpi. As expected, the lower number of late TDFs from 48 hpi onwards went hand in hand with a smaller number of functional categories. A high number of modulated transcripts of both genotypes were of unknown function.

### Microarray analysis

The transcripts identified by cDNA AFLP analysis were used to design a custom oligo-microarray for studying the response of the resistant F1 21/66 compared with the parent Teroldego and the 22/73 offspring, both susceptible to the fungus. In addition to the 278 TDFs, probes representing another 72 genes known to be involved in plant-pathogen interaction were also included. The arrays were hybridized with total RNA extracted from leaves of the three genotypes collected at 0 hpmi (control sample), 12 and 96 hpi (treated samples) (Additional file 4). These time points were chosen because they corresponded to the early and late phases of transcriptional modulation observed in the cDNA-AFLP experiments.

Comparative analysis of the treated samples versus the control sample within each genotype highlighted 93, 45 and 36 modulated genes in F1 21/66, Teroldego and F1 22/73, respectively (Additional file 5). Of the 93 modulated genes in F1 21/66, 42 showed the same profile as in the cDNA-AFLP analysis, although the sampling times were only partially overlapping.

In particular, 19 of the 93 modulated genes identified in the resistant genotype were also up-regulated in the susceptible individuals. Most of the genes in this subset belong to three categories: response to stimulus, primary metabolism and photosynthesis. A group of 57 genes were exclusively modulated in the resistant genotype. Of these, 48 were up-regulated at 96 hpi, 4 were up-regulated at 12 hpi while the remaining 5 were down-regulated at one of the time points. Some of these transcripts were assigned to the categories defense response, photosynthesis and primary metabolism, as were the common modulated genes, and the others were assigned to the main functional groups of secondary metabolism, signal transduction and transport. We also found a group of genes specifically modulated in the susceptible individuals, 13 of which were exclusively induced in Teroldego and 11 in the offspring (5 induced and 6 repressed) both at 12 hpi and at 96 hpi.

The microarray data for 9 differentially expressed transcripts, whose relative expression varied from 0.17-fold to 6.8-fold, were validated by Reverse Transcription quantitative Polymerase Chain Reaction (RT-qPCR) analysis (Additional file 6). They were selected because they were related to the resistance process and also because of a large variation in fold change between control and treated samples. As shown in Additional file 6, there was good agreement with the array data and in some cases the magnitude of change determined by RT-qPCR revealed greater differential expression, indicating that the microarray results underestimated actual changes in gene expression.

## Discussion

In contrast to *Vitis vinifera*, a species indigenous to Eurasia, American and Asian wild grapevine species are generally resistant to *Plasmopara viticola*, having co-evolved with this mildew which occurs in the same habitat. There is compelling evidence that there are diverse *P. viticola*-resistance mechanisms [3,12,14,15] and that they may rely on recognition of general elicitors or specific elicitors encoded by *Avr *genes, as demonstrated in other models [44,45].

In this study we used a combination of metabolic and transcriptional analyses to investigate *P. viticola *resistance in grapevine in a population of offspring generated by crossing Merzling (a complex hybrid from *V. vinifera *x *V. rupestris *x *V. lincecumii*) with *V. vinifera *Teroldego. This population clearly segregates for *P. viticola *resistance. The degree of individual sensitivity to the oomycete showed a distribution typical of traits controlled by a few major QTLs with dominant effects, in line with the literature [28,29,31,32].

A frequently observed defense mechanism in grapevine is the accumulation of phytoalexins belonging to the stilbene family [17-19,39]. We measured the concentration of the monomeric stilbenes and all the oligomer stilbenoids in the leaves of the entire population six days post inoculation and found a large variation both in the type and the relative quantity (profile) of the stilbenoids. Different levels of resveratrol monomers and oligomers have previously been reported in healthy grapes [23,46], but also in infected leaves where they have been linked to the genotype's susceptibility to *P. viticola *[19,47]. Estimated stilbenoid concentrations in the inoculated leaves ranged from less than 1 μg g -1 fw to more than 100 μg g -1 fw , suggesting that at least some of them were present at concentrations toxic for the pathogen (reviewed in Smith [48]). Results from activity assays using the isolated stilbenoids will allow us to draw final conclusions. Further investigation which merits being carried out, is a detailed kinetic analysis of stilbenoid accumulation and spreading of the pathogen in the infected leaves in order to corroborate the correlations emerging from this study. We performed our analysis at 6 dpi as this interval was ideal for discriminating stilbenic phytoalexin production in the different genotypes, as highlighted in Vrhovsek *et al*. [36].

Our data indirectly confirmed that *trans*-resveratrol and its glycosilated form *trans*-piceid are not *per se *very toxic against *P. viticola*, as previously demonstrated by direct assays (reviewed in Jeandet *et al*. [17]) and by analysis of grapevine genotypes with varying degrees of resistance to the oomycete [18,19]. Type of substitution and oligomerisation state appear to be of importance in determining the role of a stilbene as a phytoalexin [18,19,47]. The two resveratrol monomers were in fact found in most of the susceptible genotypes, while resveratrol oligomers accumulated almost exclusively in the resistant offspring. There were two kinds of exception: three genotypes with detectable levels of oligomers, but displaying an intermediate degree of sporulation (≥ 15%), and a group of genotypes with no detectable or very low levels of oligomers, but still resistant to *P. viticola*. Both groups of individuals represent highly interesting material for further analysis, in particular the latter group whose resistance could be ascribed to a different mechanism which does not involve the presence of viniferins.

Interestingly, with respect to both resistance trait distribution and stilbene profiles, the population included transgressive members which express the characteristic under investigation to an extent beyond the range delimited by the parents. For this reason our transcriptional analysis included the F1 21/66 genotype.

Several studies have demonstrated that *V. vinifera *undergoes strong transcriptional modulation upon *P. viticola *infection in order to prevent pathogen invasion [6,33,34], but the response seems to be more variable in the case of incompatible reactions. Very limited gene modulation has been reported in the interaction between *V. aestivalis *and *Erysiphae necator *[10], while more recently, study of the incompatible interaction between *V. riparia *and *P. viticola *revealed instead a pronounced transcriptional change [6].

To investigate gene expression response in our pathosystem we carried out a comparative analysis on resistant and susceptible individuals using a combination of cDNA-AFLP and oligo-array techniques. The microarray experiments highlighted very different behaviors in the resistant and the susceptible genotypes upon infection. There was a much higher number of modulated transcripts in the 21/66 offspring than in Teroldego and the 22/73 offspring. It should be noted that the design of the study does not allow us to extend this result to the fraction of genes which were not represented on the array.

Half of the F1 21/66 modulated genes had the same profile observed in the cDNA-AFLP experiment and they were generally up-regulated (Additional file 5). A difference was, however, seen in the timing of the modulation: gene induction was detected mainly after 12 hpi in the microarray experiment, whereas 53% of the genes were already induced at 12 hpi in the cDNA-AFLP study. This difference likely resides in the higher number of sampling times considered in the cDNA-AFLP study and in the fact that the microarray technique is less sensitive than the PCR-based cDNA-AFLP technique. A similar technical discrepancy was found in a recent study involving molecular analysis of resistance to leaf stripe in barley [49].

Of the 93 modulated genes in the resistant offspring only 19 were also induced in the susceptible individuals. This class includes genes encoding for proteins involved in transcription and translation activation, namely an elongation factor 1-alpha [DFCI:TC96066] and a pentatricopeptide repeat-containing protein [DFCI:TC91629], and for a phase change-related protein [GenBank:JG391699, DFCI:TC93391] and a lipid transfer protein [DFCI:TC90421] activated in other plant-pathogen interactions [50,51]. Their early up-regulation, within 12 hpi, suggests metabolic reprogramming and plant defense response following recognition of general elicitors in both resistant and susceptible genotypes.

Of special interest were 57 genes exclusively modulated in the resistant genotype. Given the functional categories and, in some cases, the specific genes affected by the oomycete, we presume that the resistance mechanism observed in our study is quite similar to that found in *V. riparia *following *P. viticola *infection [6].

Genes encoding for recognition and signal transduction components, such as two receptor-like protein kinases [DFCI:TC80277, GenBank:JG391865] and one TIR-NBS receptor [DFCI:TC98959], were slightly activated. A calcium-dependent protein kinase [DFCI:TC79194] was also specifically induced in the resistant offspring suggesting that this secondary messenger may play a role in the defense response. A major role, however, seems to be played by ethylene as a signaling molecule. Several transcripts involved in ethylene biosynthesis [DFCI:TC98757, DFCI:TC89222, DFCI:TC77376, DFCI:TC75061], as well as downstream ethylene responsive factors [DFCI:TC92107, DFCI:TC89392], appeared to be induced. Interestingly, we detected transcriptional activation of genes encoding for a *V. vinifera *osmotin-like protein [GenBank:Y10992] and a β1,3-glucanase [GenBank:AJ277900], which belong respectively to class 5 and class 2 pathogenesis-related proteins. Several studies have proved that ethylene modulates grapevine PR-5 and PR-2 genes [52] and have shown the role these play in resisting biotrophic and necrotrophic pathogens [53]. Consistent with previous reports regarding *P. viticola*-infected grapevine leaf discs [12], we also observed accumulation of a PR1 [GenBank:AJ536326] and a PR10 [GenBank:AJ291705] transcript at 96 hpi.

Resveratrol accumulation is strictly controlled at the transcriptional level by regulation of the steady state of stilbene synthase transcripts [54], both during development [23] and under elicitation [24-27]. However, no transcriptional regulators have been identified so far. As expected, we found two isoforms of stilbene synthase, [GenBank:S63225] [55] and [GenBank:X76892] [56], which were activated in the resistant individual at 96 hpi. On the other hand, no modulation was observed in the susceptible genotypes. The timing of the induction is consistent with our biochemical results and with the literature [47]. In particular, strong up-regulation of the isoform [GenBank:S63225] (12 times that detected in RT-qPCR), between 12 and 96 hpi, is indeed compatible with the complex profile of viniferins accumulated in the resistant offspring at six days post infection.

Interestingly, cDNA-AFLP analysis revealed induction of the expression of two peroxidase genes [DFCI:TC81349, DFCI:TC56380] in the resistant offspring at 24 hpi. Peroxidases are known to catalyse oxidation of *trans*-resveratrol in the presence of H_2_O_2_, giving rise to a resveratrol radical which then oligomerizes to form the stilbenoid oligomers [57,58].

We found three other induced genes belonging to the phenylpropanoid metabolism, encoding for a caffeoyl-CoA O-methyltransferase [GenBank:Z54233], a flavonoid 3', 5'-hydroxylase [GenBank:CF404908] and a dihydroflavonol reductase [GenBank:X75964]. Although we did not check accumulation of monolignols and proanthocyanidins in the infected leaves, they are known to play a role in the plant's defense response. Monolignols are essential for cell wall reinforcement [59] and proanthocyanidins are toxic compounds for pathogens [60,61].

The defense response in biotrophic interactions also involves primary metabolism reprogramming [6]. In our study, several genes which could be associated with protein degradation appeared to be induced by *P. viticola *in the resistant genotype, as reviewed previously for other plant-pathogen interactions [62].

Interestingly, a ubiquitin E3-ligase with RING-H2 domain [DFCI:TC101906] and a ubiquitin protein [DFCI:TC85973] were induced upon infection, as observed in *V. riparia *[6]. Many other genes encoding for catabolic enzymes of proteins (carboxypeptidases, aminopeptidases) and carbohydrates (amylases) were also up-regulated at 96 hpi.

Most of the 26 modulated genes specific to susceptible individuals turned out to be induced (74%) but did not exhibit a coherent expression profile in either susceptible genotypes. This analysis does not, therefore, allow us to draw conclusions about the mechanisms underlying grapevine-*P. viticola *compatibility, also because 16 of these genes showed the same cDNA-AFLP profile in the resistant genotype.

Among the modulated genes, we found 15 genes whose modulated expression had no common rule in the resistant versus the two susceptible genotypes. This group contained genes encoding for a plastidic aldolase [GenBank:JG391820] and for photosynthetic proteins such as chlorophyll a-b binding proteins [DFCI:TC93431, GenBank:JG391764, DFCI:TC84281, DFCI:TC73356], a cytochrome b [DFCI:TC78321] and a ribulose 1-5-bisphosphate carboxylase/oxygenase activase [GenBank:JG391868]. Most of the genes were already down-regulated in Teroldego at 12 hpi and highly activated in the resistant offspring, mainly at 96 hpi. Down-regulation of photosynthesis-related genes following pathogen infection in susceptible genotypes during compatible interactions has already been widely reported [10,33,63-65]. Up-regulation of the photosynthetic genes in resistant genotypes, as reported here, has been described in only a few cases [66]. This could be an alternative strategy adopted by the cell to gain energy for defense response, as opposed to induction of invertase activity previously described in the case of *P. viticola *infection [6].

## Conclusions

This work reports a biochemical and transcriptomic analysis of downy mildew resistant and susceptible individuals selected from a grapevine crossing population (Merzling × Teroldego) which segregates for resistance and stilbenoid content traits.

A strong negative correlation between the concentrations of stilbenoid viniferins in the leaves and the progress of infection was demonstrated. Moreover, a comprehensive transcriptome profiling of resistant and susceptible individuals of the cross following infection led to the identification of a set of genes specifically modulated in the resistant genotype which should be taken into account in future breeding programs.

## Methods

### Plant material, inoculum and plant infection methods

An interspecific population derived from Merzling (M) (complex hybrid of *V. vinifera *descending from *Vitis rupestris *and *Vitis lincecumii*) × *V. vinifera *cv Teroldego (T) was characterized for resistance to *P. viticola *and for accumulation of stilbenoid compounds upon infection. The cross was developed at the Fondazione Edmund Mach and consisted of 255 progeny plants. Of the 255 F1 individuals, those selected were replicated annually by grafting wood cuttings onto rootstock KOBER 5BB. The plants were grown in 1L pots filled with soil:sand:peat:vermiculite (3:1:3:3, v/v) in a greenhouse at 25°C/20°C day/night temperature, with a 16 h photoperiod and relative humidity (RH) of 70 ± 10%. Sporangiophores of *P. viticola *(Berk. and Curt) Berl. et De Toni were collected from infected leaves of *V. vinifera *cv Pinot Gris plants by brushing the white mould present on the underside of the leaves in cold bidistilled water. Fully expanded leaves of 8 to 10 week old grafted plants were inoculated by spraying a conidial suspension of of 10^4^/10^5^ spores/ml onto the abaxial leaf surface and were kept overnight in the dark in a growth chamber at 24°C with 80% RH. The infected plants were then transferred to the greenhouse and kept in the same conditions as described above. Mock-inoculated plants were obtained by spraying distilled water in the greenhouse.

Plants were organized on the basis of experimental design specific to each analysis (phenotypic evaluation, stilbenoid analysis, gene expression analysis).

### Phenotypic evaluation of resistance to *P. viticola*

The parental lines plus 104, 87 and 86 of the 255 F1 individuals were scored for resistance to *P. viticola *in 2005, 2006, 2007 respectively.

Plant reaction was scored as presence or absence of visible necrosis at ten days post infection (dpi). The extent of sporulation was assessed by visually estimating the percentage area of sporulation (% Sp) on the lower leaf surface on all infected leaves of all replicates according to [67]. A mean value and a standard error were calculated for each individual. Magnitude of plant reaction and level of sporulation per individual were simultaneously rated by a visual index, the OIV452 descriptor, recommended by the Office International de la Vigne et du Vin [37]. Categorical values from 1 (the most susceptible) to 9 (the most resistant) were assigned based on absence or presence of visible necrosis and its size, as well as on the extent of sporulating area: 1: sporangiophores densely cover the whole leaf area, diffuse chlorosis, absence of necrosis; 3: predominating patches of dense sporulation, chlorotic areas, absence of necrosis; 5: patches of sparse sporulation equally intermixed with asymptomatic areas, necrotic flecks underneath sporulating areas; 7: small spots with sparse sporangiophores, concentric development of necrotic lesions with HR; 9: absence of sporangiophores, small necrotic spots with HR. Even numbers were used to describe intermediate categories.

The absence of *P. viticola *symptoms was confirmed on all the leaves of the control plants.

Normal distribution of sporulation values was assessed by the One-Sample Kolmogorov-Smirnov test applied to the % Sp values, square root transformed (RADQ S) using the Statistica data analysis software version 6 (StatSoft, Tulsa, OK).

### Analysis of stilbenoid content

The second and the third leaf from the apex of one biological replicate for each genotype were collected at 6 dpi and at 0 hpmi during the 2005 harvest. All the leaves collected were stored at -20°C until analysis. Sample preparation and the conditions for HPLC-DAD-MS analysis were the same as described in Vrhovsek *et al*. [36]. The stilbene monomers and stilbenoid oligomers were identified by comparing the retention time, MS and UV spectra with those of authentic standards, and quantified by UV-VIS detection at 280 nm and 310 nm using the external standard method. *Trans*-Resveratrol, *trans*-piceid and IS (*trans*-4-hydroxystilbene) monomers were quantified with UV-VIS detection at 310 nm. Dimers ((+)-*E-*ε-viniferin, *Z*- and *E*-ω-viniferin, ampelopsin D and quadrangularin A), trimers (*Z*-miyabenol C and *E-*miyabenol C and α-viniferin) and tetramers (isohopeaphenol, ampelopsin H and vaticanol-C-like isomer) were quantified according to the calibration curves of the isolated compounds. Pallidol was expressed as ampelopsin H, *trans*-pterostilbene was expressed as the equivalent of *trans*-resveratrol. Due to the coelution of vaticanol-C-like isomer and ampelopsin H the sum of both compounds was expressed as ampelopsin H, due to the coelution of *Z+E*-miyabenol C the sum of both compounds was expressed as *Z*-miyabenol C, and due to the coelution of *Z+E*-ω-viniferin the sum of both compounds was expressed as *E*-ω-viniferin. All concentrations are expressed as mg/kg of fresh weight (fw).

### cDNA-AFLP analysis

Leaves for the analysis (the second and third from the apex) were collected from five biological replicates each of F1 21/66 and Merzling at 12, 24, 48 and 96 hpi with *P. viticola *and at 0 hpmi (C) in the summer of 2005, immediately frozen in liquid nitrogen and stored at -80°C. Total RNA was extracted from a pooled sample of the second and third frozen leaf according to Moser *et al*. [68], quantified by Nanodrop 8000 (Thermo Scientific) and checked for quality using an Agilent 2100 Bioanalyzer (Agilent Technologies).

Double-stranded cDNA synthesis and cDNA-AFLP procedures were as previously described in Polesani *et al*. [33], starting from 2 μg of total RNA and using BstYI and MseI as restriction enzymes. A total of 128 selective amplifications were carried out with^33^P-labeled *Bst*YI primers containing one extra selective nucleotide per primer. The amplification products were separated and the gels were scanned as described in Polesani *et al*. [33]. Differentially expressed transcripts relating to inoculated and control samples were identified by visual inspection of autoradiographic films and their profiles were visually scored and were assigned the term U to fragments 'up-regulated in infected samples', D to those 'down-regulated in infected samples' and S to those with 'the same profile after infection or water-spray treatment' (Additional file 3). To validate the reproducibility of the cDNA-AFLP data, the selective amplification reaction of 6 primer combinations was replicated twice starting from two independent pre-amplification products. Bands corresponding to differentially expressed transcripts were excised from the gels and eluted in 100 μl of sterile bidistillated water. An aliquot of 5 μl was used as a template for re-amplification with non-labeled primers identical to those used for selective amplification.

PCR products were purified by adding 1.5 μl of exonuclease-phosphatase (ExoSAPIT, Amersham) to each 5 μl of PCR product which was incubated at 37°C for 45 min, then at 75°C for 15 min and then directly sequenced.

### Sequence analysis and annotation

Sequences were analyzed by homology searching with BLAST [69] against the following databases: EST database at NCBI [70], DFCI Grape Gene Index (release 6.0) [71], IASMA Grape Genome database (release 3.0) [72], RefSeq blast database at NCBI [73] and UNIPROT [74]. Blast results (blast-n: E-value < 10^-10^, blast-x: E-value < 10^-6^) with GO associated terms were analyzed by the 'ARGOT' tool for annotation developed in-house [75] with high confidence (Id > 80%). Automatic annotation results were manually inspected and integrated with GO 'biological process' terms supported by evidence from the literature. Finally, sequences were assigned to functional categories.

Sequence data have been deposited at NCBI's EST database [70] and are accessible through GenBank, accession numbers: JG391664-JG391941.

### Combimatrix array design

Genes considered for representation on microarrays included those containing the 278 cDNA-AFLP fragments that exhibited differences in band intensity according to genotype/treatment and gave good quality sequences, in addition to the 72 coding for proteins which are reported in the literature as having possible or demonstrated roles in pathogen defense. For most of the sequences, two probes twice-spotted were designed into different regions, while in the case of non-oriented sequences, two probes were designed into each direction with the suffix 'RC' added to the name of the probe corresponding to the Reverse Complement strand. A total of 1530 probes were synthesized onto each sector of a CustomArray 4×2240 microarray slide (Combimatrix Corp., WA). Negative control probes from viruses and bacteria (Combimatrix Corp., WA) and four putative housekeeping genes were also included on the array.

### Hybridization and microarray analysis

Hybridization probes were made from 18 total RNA preparations representing two biological replicates of the leaves of F1 21/66, F1 22/73 and Teroldego inoculated with *P. viticola *and collected at 12 and 96 hpi, or sprayed with water (C).

Total RNA (1 μg) was amplified using the Amino Allyl MessageAmp™II aRNA Amplification kit (Ambion, USA) and the resulted amminoallyl-aRNA was conjugated to a fluorescent label (Cy-5). The purified labeled aaRNA was quantified by spectrophotometry (ATI Unicam) and 2 μg were hybridized to the custom Combimatrix array according to the manufacturer's directions. Each hybridization was repeated three times. Pre-hybridization, hybridization, washing and imaging were performed according to the manufacturer's protocols [76].

The arrays were scanned with a ScanArray4000XL (Perkin Elmer, USA) and TIF images were exported to MicroArray Imager 5.8 (Combimatrix, USA) for densometric analysis. Microarray data were analyzed according to the procedure described in [49] with some modifications. Briefly, spot flagging and visual inspection of the images was carried out in order to exclude bad spots (based on spot saturation and heterogeneity). Raw data were analyzed and negatively flagged spots were excluded from further analysis by assigning them a zero weight. Only probes with a signal intensity of at least 500 fluorescence units [77] for all biological replicates were considered for further analysis. Scaling normalization was performed using *Actin *and *Ufgt *(UDP-glucose:flavonoid 3-O-glucosyltransferase) as reference genes. The normalized median intensity values were Log2-transformed.

For each dataset, a Pearson correlation test was performed on the normalized Log2-transformed values in order to assess the variability within technical and biological replicates. Datasets from each individual were analyzed independently after calculating the mean expression value from normalized values of technical replicates for each probe (due to the range of Pearson coefficients obtained) (Additional file 4). Before statistical analysis, a mean value was calculated from normalized values of hybridization technical replicates (Log2-mean value). For each individual, the normalized values were organized in three groups corresponding to the harvesting time points for comparison. Three datasets with high quantities of significantly differentially expressed genes were identified by running a Significance Analysis of Microarrays (SAM) multiclass comparison [78] using a TIGR Multiexperiment Viewer [71] with a False Discovery Rate (FDR) < 5% imposed, as in [6]. SAM output was further restricted to genes with a change in mRNA expression of 1.5-fold or greater in at least one of the two analyzed expression points. For those genes in which two oligos were found significantly differentially expressed, a mean value was calculated from the median intensity values at each time point (Additional file 5).

Expression data have been deposited in NCBI's Gene Expression Omnibus [79] and are accessible through GEO Series accession number GSE28851.

### Real-time RT-PCR analysis

Total RNA for Reverse Transcription quantitative Polymerase Chain Reaction (RT-qPCR) were the same as those used for the array hybridizations. For each time point, RNA was initially treated with RNase free-rDNaseI (Ambion) and subsequently used for first strand cDNA synthesis using the Superscript™ III Reverse Transcriptase kit (Invitrogen) according to the manufacturer's instructions. Amplification was performed using SYBR Green PCR master mix, as described in [23], using gene-specific primers designed within the same genomic region where the oligos for microrray analysis were localized (see Additional file 6 for sequences). Cycling conditions were: 50°C for 2 min, 95°C for 2 min, then 40 cycles of 95°C for 15 sec and 60°C for 1 min. Triplicate quantitative assays were performed with an ABI PRISM 7000 Sequence Detection System (Applied Biosystem, Foster City, CA). Raw data were analyzed with ABIPRISM 7000 DS software to extract Ct values. Baseline-corrected data were imported into the LinRegPCR software to calculate reaction efficiency [80,81]. The relative expression of each gene (target) was then calculated according to Pfaffl's equation [82] using *Actin *for normalization (reference) and the water-sprayed control as calibrator sample (control), which represents 1X expression of the gene of interest. The overall standard error (SE) of the mean normalized expression was obtained by applying the error calculation based on Taylor's series as developed for REST^© ^software [83].

## Abbreviations

cDNA: Complementary DNA; EST: Expressed Sequence Tag; GEO: Gene Expression Omnibus; HPLC-DAD-MS: High Performance Liquid Chromatography - Diode Array Detection - Mass Spectrometry; NCBI: National Center for Biotechnology Information; RT-qPCR: Reverse Transcription quantitative Polymerase Chain Reaction; SAM: Significance Analysis of Microarrays; TC: Tentative Consensus.

## Competing interests

The authors declare that they have no competing interests.

## Authors' contributions

GM carried out the cDNA-AFLP and microarray experiments (including the Combimatrix array design), performed microarray statistical analyses, helped with grapevine infections, collaborated on conception of the study and wrote the manuscript. UV performed the biochemical analyses. LZ carried out grapevine infections and MS developed the M × T cross and provided the woody cuttings. AC helped with the sequence analysis and annotation. FM collaborated on conception of the study and on critical interpretation of the biochemical results, and revised the manuscript. MD collaborated on experimental design and is responsible for the Functional Genomics Center (University of Verona) where the hybridization experiments were carried out. RV conceived the study and revised the manuscript. CM collaborated on conception of the study and in writing the manuscript. All authors read and approved the final manuscript.

## Supplementary Material

Additional file 1 **Quantification of the 16 stilbenoids in the Merzling (M) × Teroldego (T) cross after infection with *P. viticola***. The file contains quantification (μg/g fw) and percentages (%) of the 16 stilbenoids identified in a pooled sample of the second and third leaves of the shoot (2+3) of one replicate (r) of the 106 individuals of the cross at 6 dpi. Individuals were assigned to three groups according to stilbenoid production (high producers, low producers, non-producers). Phenotypic data collected during the 2005 harvest (percentage area of sporulation (% Sp) on the lower side of leaves, hypersensitive response (HR) and OIV452 categories) are reported in the last three columns. ID: numeric code assigned to each genotype. n.d.: phenotype not detected due to the absence of replicated plants for phenotypic analysis Click here for file

Additional file 2 **Profiles of the 16 stilbenoids in the 18 high producers of the Merzling (M) × Teroldego (T) cross**. The figure shows the percentage (%) of each stilbenoid identified by the HPLC-DAD-MS analysis in the infected leaves of the 18 high producers. Click here for file

Additional file 3 **List of transcript derived fragments (TDFs) isolated in Merzling and in F1 21/66 after infection with *P. viticola *by cDNA-AFLP analysis**. The file contains a complete list of TDFs showing differential expression, visually estimated by comparing the intensity of the bands in inoculated and control samples of Merzling and F1 21/66. For each TDF is reported: i) the identifier (ID) (1, 2, 3, 4 correspond to A, T, C, G nucleotides used for the selective amplification, BC/BT = BstT0/BstC0, M = Mse0); ii) the GenBank accession number; iii) the expression profile at 12, 24, 48, 96 hpi and at 0 hpmi (C); the abbreviations S, U, D stand for 'the same profile after infection or water-spray treatment', 'up-regulated in infected samples' and 'down-regulated in infected samples', respectively; -- = band not detected due to absence of amplification; iv) the length; v) results of the annotation process: description of a putative function (if available) together with the EST/TC accession numbers, gene and protein accession numbers obtained by blastn/x against EST database at NCBI [70], DFCI Grape Gene Index [71], IASMA Grape Genome database (release 3.0) [72], RefSeq database [73] and UNIPROT database [74]. Click here for file

Additional file 4 **Results of microarray analysis performed on resistant and susceptible individuals of the Merzling (M) × Teroldego (T) cross after infection with *P. viticola***. The file contains the results of significance analysis of microarrays (SAM) multiclass comparison obtained for each genotype (F1 21/66, Teroldego, F1 22/73). For each probe is given: i) the oligo identifier (ID); ii) the accession number of the spotted sequence; iii) the Log2 mean expression value of three technical replicates (Log2-mean value) at 0 hpmi (C), at 12 and at 96 hpi; iv) the change in mRNA expression in treated vs control samples (fold change) calculated as 2 EXP (Log2-mean value of treated sample - Log2-mean value of control sample); v) the q-value indicating the False Discovery Rate (FDR) (bold values are those below the selected threshold of 5%). Click here for file

Additional file 5 **Complete list of significantly modulated genes in F1 21/66, Teroldego and F1 22/73 after infection with *P. viticola***. The file contains the list of transcripts exhibiting statistically significant differential expression with a False Discovery Rate (FDR) < 5% and a fold change greater than 1.5. For the F1 21/66 genotype both cDNA-AFLP and microarray results are reported (light gray indicates agreement between cDNA-AFLP and microarray profiles). For each transcript is given: i) the TDF identifier or the abbreviation of the gene code (ID); ii) the accession number of the spotted sequence; iii) a description of the protein function and the functional category; iv) the cDNA-AFLP profile in F1 21/66; v) the fold change at 12 and 96 hpi in F1 21/66, Teroldego and F1 22/73, as obtained by microarray analysis. Click here for file

Additional file 6 **Real-time RT-PCR validation of the expression profiles of nine *P. viticola*-responsive genes**. differences between the *P. viticola*-inoculated and control conditions as measured by Reverse Transcription quantitative PCR (RT-qPCR) and by microarray for nine *P. viticola*-responsive genes in three individuals of the cross. For each transcript is given: i) the TDF identifier or the abbreviation of the gene code (ID); ii) a description of the protein function; iii) the sequences of the primers used for the amplification (sequences for *Actin *are from Gatto *et al*. [23]; iv) the fold change (FC) at 12 and 96 hpi in F1 21/66, Teroldego and F1 22/73 as obtained by array hybridization experiments and RT-qPCR. SE is the overall standard error of the mean normalized expression value. Click here for file
